# Coronavirus Disease Pandemic Effect on Medical-Seeking Behaviors Even in One Resource-Competent Community: A Case Controlled Study

**DOI:** 10.3390/ijerph191710822

**Published:** 2022-08-30

**Authors:** Fang Wang, Jin-Ming Wu, Yi-Chieh Lin, Te-Wei Ho, Hui-Lin Lin, Hsi-Yu Yu, I-Rue Lai

**Affiliations:** 1 Department of Nursing, National Taiwan University Hospital, Taipei 100, Taiwan; 2 Department of Surgery, National Taiwan University Hospital and National Taiwan University College of Medicine, Taipei 100, Taiwan; 3 Department of Surgery, National Taiwan University Hospital Hsin-Chu Branch, Hsinchu 300, Taiwan; 4 Department of Public Health, College of Health Sciences, Kaohsiung Medical University, Kaohsiung City 807, Taiwan

**Keywords:** health-seeking behavior, COVID-19, appendicitis, surgical care

## Abstract

(1) Background: The coronavirus disease 2019 (COVID-19) pandemic had overwhelming impacts on medical services. During its initial surge, Taiwan was unique in maintaining its medical services without imposing travel restrictions, which provided an ideal environment in which to test if the fear of becoming infected with COVID-19 interfered with health-seeking behavior (HSB). We tested this hypothesis among adults with acute complicated appendicitis (ACA). (2) Methods: Adults with acute appendicitis were enrolled between 1 January and 30 June 2020 (COVID-19 period). The first two quarters of the preceding 3 years were defined as a historical control group. Outcome measures included the rate of ACA and the number of hospital stays. (3) Results: The COVID-19 era included 145 patients with acute appendicitis. Compared to the historical control (320 patients), the COVID-19 era was significantly associated with a higher length of symptom duration until presentation to the emergency room within >48 h (17.2% vs. 9.1%, *p* = 0.011), a higher incidence of ACA (29.7% vs. 19.4%, *p* = 0.014), and a longer length of hospital stays (5.0 days vs. 4.0 days, *p* = 0.043). The adjusted models showed that the COVID-19 period had a significant relationship with a higher rate of ACA (odds ratio (OR) = 1.87; 95% confidence interval (CI): 1.23–2.52; *p* = 0.008) and longer length of hospital stays (OR= 2.10; 95% CI: 0.92 to 3.31; *p* < 0.001). (4) Conclusions: The fear of COVID-19 may prohibit patients from seeking medical help, worsening their clinical outcomes. The surgical community should take action to provide scientific information to relive mental stress.

## 1. Introduction

Health-seeking behavior is described as “steps taken by a patient who perceives a need for help when he or she tries to solve a medical disease” [1]. In other words, health-seeking behavior is the behavioral element of healthcare utilization and is conceptually associated with clinicodemographics, socioeconomic status, access to healthcare institutes, and the views and experiences of both the patient and medical provider [2,3]. Andersen developed a serial behavioral model to elucidate the mechanisms associated with healthcare utilization [2,4], defined as the quantity of people using healthcare services and estimated by costs and medical visits [5].

The new variant of severe acute respiratory syndrome coronavirus disease 2 (SARS-CoV-2), also known as coronavirus disease 2019 (COVID-19), first broke out in China in December 2019 and rapidly spread worldwide [6]. As of 30 September 2020, 33.83 million confirmed cases and >1.01 million deaths have been cumulatively recorded worldwide since the start of the pandemic according to the World Health Organization statistics [7]. The highly contagious potential of COVID-19 deepened the strain on healthcare institutes and resulted in reduced healthcare utilization worldwide under a limited supply of medical resources. Furthermore, patients with severe illnesses might be unwilling to visit a highly contagious hospital due to their fear of being infected by the virus [8].

Acute appendicitis is not only one of the most common causes of acute abdominal diseases in adults [9], but also one of the most common reasons for general emergency surgery worldwide [10]. With the advancement of minimally invasive surgery, most patients can recover early after a timely appendectomy [11]. However, the COVID-19 pandemic became a barrier to timely surgical treatment, which increased the ACA rate in countries with compromised medical supplies [12,13].

Taiwan is very close to the coast of China and was predicted to be likely to experience a high number of cases in 2020 due to the many flights that occur between the countries. However, Taiwan surprisingly controlled its sporadic outbreaks of COVID-19 well by implementing several public health responses [14] and did not suffer a shortage of medical resources. As a result, it provides a unique environment in which to validate if health-seeking behavior may have interfered with clinical outcomes even in a resource-unlimited setting. In this study, we hypothesized that the mental panic caused by the COVID-19 pandemic may have worsened the clinical outcomes of patients. Thus, we aimed to analyze the rates of uncomplicated/complicated appendicitis during the COVID-19 pandemic and compare them with those of previous years.

The rest of this paper is organized as follows. Section 2 is the Materials and Methods, Section 3 is the Results, Section 4 is the Discussion, and Section 5 is the Research Conclusions.

## 2. Materials and Methods

A retrospective study was conducted to review the records of adult patients with acute appendicitis (≥20 years) in one academic center during the COVID-19 epidemic period from January to June 2020. Next, patients with acute appendicitis from the same months, January to June, in 2017, 2018, and 2019 were considered as the control group. The same interval every year was analyzed because a previous study demonstrated that the incidence of acute appendicitis was associated with seasonal variation [15]. The exclusion criteria were being pregnant or pathological findings of appendiceal tumors. 

The data analyzed included clinical demographics such as age, gender, body mass index (BMI), residence in Taipei/New Taipei City, body temperature measured at the emergency room (ER), white blood-cell count (WBC), and the time interval from symptom onset to ER arrival. The weighted Charlson comorbidity index (CCI) score was used to account for the comorbidity burden [16,17]. Further, daily confirmed COVID-19 cases were recorded to represent the severity of the pandemic. The primary outcome measures were the occurrence of ACA defined as abscess observed in computed tomography, presence of appendiceal perforation determined by surgical documentation, or a description of gangrenous appendicitis assessed by pathological reports [10]. The secondary outcome measure was inpatient length of stay.

Furthermore, the trend of the total number of surgeries (elective and emergent operations) during the study periods was analyzed to reflect the overall impact of COVID-19 on surgical patients seeking medical services. 

Statistical analyses were performed using the Statistical Package for the Social Sciences (SPSS) version 26.0 software (IBM SPSS 26.0, Armonk, NY, USA). Continuous variables (age, body mass index, time from ER visit to surgery, white blood count, and length of hospital stays) were presented as medians with interquartile ranges (IQRs), and categorical variables (gender, category of Charlson comorbidity index score, residence in Taipei/New Taipei City, category of duration of symptoms until presentation to ER, body temperature > 38 degrees Celsius, and appendectomy performed) were expressed as numbers (percentages). Categorical variables were compared using the Chi-square test or Fisher’s exact test (the numbers were <5). Continuous variables between groups were compared using the Mann–Whitney U test. The binary logistic regression model was used on associated variables to determine the odds of ACA occurrence. Furthermore, a linear regression model was created to predict the length of hospital stays. Statistical significance was assumed at *p* < 0.05.

## 3. Results

During the 26-week COVID-19 study period, 145 patients with acute appendicitis were treated in our institute (Table 1). No patient was diagnosed with COVID-19. Among them, 43 (29.7%) patients had ACA. The rates of appendectomy in the ACA and non-ACA groups were 83.7% and 98.0%, respectively. Patients with ACA had a significantly higher rate of body temperature > 38 °C (46.5% vs. 26.5%, *p* = 0.019) and a significantly higher rate of symptom duration until presentation to the ER within >48 h (20.9% vs. 8.8%, *p* = 0.001) in comparison with the non-ACA group. No differences were observed in the median age, gender, BMI, or CCI score category between adults with ACA and non-ACA.

**Figure 1:**
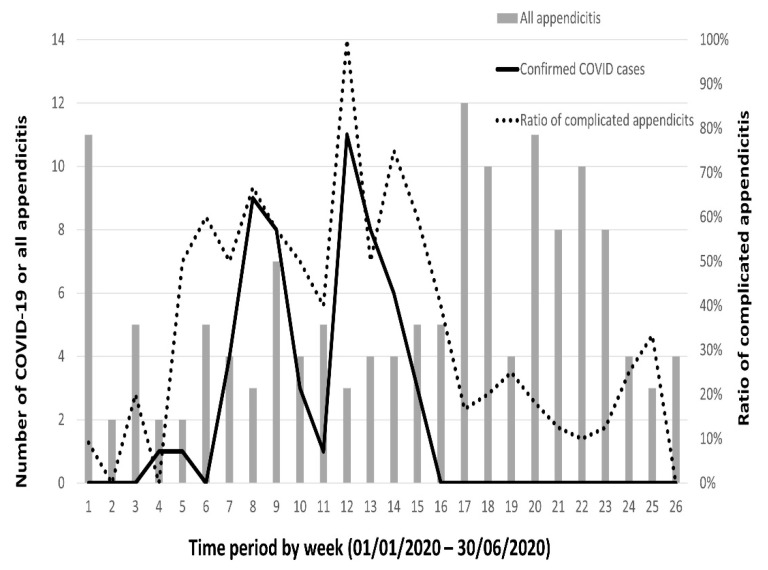
Weekly statistics for the number of adult patients with acute appendicitis (dotted vertical bar), the number of cases of complicated appendicitis (solid vertical bar), and the number of confirmed COVID-19 cases in Taiwan (dashed line).

To determine the impact of the COVID-19 pandemic on the severity of appendicitis, Figure 1 shows the association between the number of confirmed COVID-19 cases in Taiwan and the distribution of patients with acute appendicitis. The majority of COVID-19 cases were diagnosed from week 6 to 16. The ACA ratio during the same period was also increased.

For comparisons during the COVID-19 period, a control cohort comprising 320 patients was identified over 18 months between 2017 and 2019 (Table 2). No differences were observed between the two groups in terms of median age, gender, median BMI, category of CCI score, and residence in Taipei/New Taipei City. The median WBC count (10.8 vs. 9.4 × 10^9^/L, *p* = 0.010) and rate of symptom duration until presentation to the ER within >48 h (17.2% vs. 9.1%, *p* = 0.011) were significantly higher during the COVID-19 period compared to the control period. Overall, a higher incidence of ACA (29.7% vs. 19.4%, *p* = 0.014) and a longer median length of hospital stays (5.0 days vs. 4.0 days, *p* = 0.043) were observed during the COVID-19 period.

To validate the association between the COVID-19 period and ACA onset, one binary multivariate model was developed to predict ACA occurrence (Table 3), showing that both the COVID-19 period (odds ratio (OR) = 1.87; 95% confidence interval (CI): 1.23–2.52; *p* = 0.008) and symptom duration until presentation to the ER within >48 h (OR = 1.70; 95% CI: 1.06–2.36; *p* = 0.044) were significantly associated with ACA development. Further, the adjusted linear regression model was used to predict the length of hospital stays (Table 4), demonstrating that both CCI scores of >2 (coefficient = 2.51; 95% CI: 1.25 to 3.16; *p* < 0.001) and the COVID-19 period (coefficient = 2.10; 95% CI: 0.92 to 3.31; *p* < 0.001) were significantly associated with a longer length of hospital stays.

**Figure 2:**
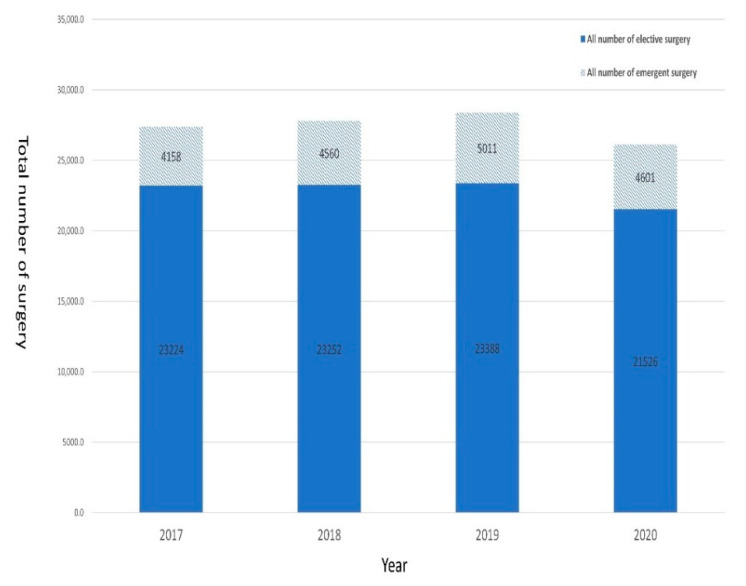
The trend of total number of surgeries (elective and emergent operations) during the study periods.

On the other hand, the trend of the total number of surgeries during the study periods is shown in Figure 2. The mean numbers of elective operations in the control period and the COVID-19 period were 23,288 and 21,526 (*p* = 0.180), respectively, whereas the mean numbers of emergent operations in the control period and the COVID-19 period were 4576 and 4601 (*p* = 0.650), respectively. Although both were not statistically significant, the COVID-19 pandemic caused a more significantly decreased number of elective operations compared to emergent operations. This implies that the COVID-19 pandemic not only changed the behavior-seeking surgical services of patients without emergent needs but also worsened the surgical outcomes of patients with emergent needs. 

## 4. Discussion

The COVID-19 pandemic was the most concerning health problem in 2020 because it caused not only health disorders but also extensive restrictions on people’s daily lives worldwide. During our study period, Taiwan avoided the worst effects of the pandemic due to its implementation of a broad public health infrastructure and domains of effective screening, isolation/quarantine, and facial mask use [18]. Although Taiwan’s government did not implement travel restrictions and its medical services were sufficient, this study demonstrated that there were fewer elective operations compared to emergent operations due to the pandemic. Further, a higher rate of ACA and a longer length of hospital stays in patients with acute appendicitis during the COVID-19 period in comparison to the control period were noticed. Notably, the number of confirmed COVID-19 cases was positively correlated with the ACA rate. Therefore, patients’ fear of being contaminated by COVID-19 influenced their health-seeking behavior and worsened their clinical outcomes.

In our study, symptom duration from ER presentation to >48 h after was found to be significantly associated with ACA, which was similar to the results of a previous report [19]. Prolonged appendicitis without prompt treatment might lead to more severe inflammation of the appendiceal wall and result in perforation or abscess formation [20]. Furthermore, a more significant association was observed between patients with acute appendicitis during the COVID-19 period and ACA as compared with that in the last 3 years. Our findings coincided with those of published reports on children [12,21] and adults [22].

In addition to the impact of clinical and socioeconomic determinants on outcomes, poor health-seeking behavior has been proven to increase morbidity and mortality [23]. Patients’ behaviors should be understood at this level in order to improve their clinical outcomes [24]. Based on our findings, one epidemic communicable disease could interfere with the need to seek out medical services even in a resource-competent community. We hypothesize that fake news or misinformation on social media may have caused people to feel excessive fear, diminishing their willingness to travel and seek medical services [25]. Conversely, scientific information and medical knowledge can help to improve health-seeking behavior, which in turn leads to a healthy condition [23]. While social media can rapidly disseminate information, the spreading of false information can confuse and distract people. Therefore, reliable evidence is a cornerstone for promoting health awareness and implementing health policies, especially during the COVID-19 pandemic. Moreover, as educated scientists and physicians should be the leaders in delivering heath-related information to the public [25,26], governments should implement anti-misinformation actions to minimize possible adverse effects.

Although healthcare systems, disparities of medical supplies, and socioeconomic status are the main barriers to medical services [27,28], the extensive health crisis that took place during the COVID-19 pandemic may become an additional obstacle in providing timely surgical intervention, a key aspect of any healthcare system with both elective and emergency procedures. To overcome the shortage of medical supplies, healthcare systems should aim to rapidly prioritize available resources and adopt new policies to deliver clinical services [29,30]. Although surgical management varies widely according to the regional culture and healthcare system, each institution has customized plans to maintain appropriate surgical services to are sufficiently protect surgical staff.

Based on our experiences, surgical need declined during the COVID-19 pandemic even if the outbreak was not severe in Taiwan. We allocated the members of surgical teams to other departments in order to prevent infection and enable better care for patients with COVID-19. During the study period, more than 24,000 man-hours of labor were provided by our surgical teams. With this help, the workload of the departments in charge of care relating to COVID-19 was partially relieved. 

This study had some limitations. The study design was retrospective and it was conducted in one universal healthcare system, which alleviated part of the barrier of access to healthcare services. Second, the period from 1 January to 30 June 2020 was selected because the spread of COVID-19 was more severe during this period. The use of different study periods might lead to different results. Third, the referral policy was associated with delayed medical visits but was consistent during the study period in Taiwan. Some studies addressed the fact that general practitioners preferred to offer medical treatment for patients with acute appendicitis without referring them to the ER [31]. Based on our findings, the number of patients with acute appendicitis (145) was higher than the average number during the control period (around 107). Therefore, we considered that the number of patients who were not referred for or who received no medical treatment to be rather low.

## 5. Conclusions

Based on our findings, the COVID-19 era had a significant relationship with a delayed presentation to the ER and led to an increased rate of acute complicated appendicitis in a community with normally functioning medical services and no travel restrictions. Medical professionals play a key role in delivering accurate information and knowledge, helping to eliminate panic, and encouraging patients to pursue medical attention promptly. With the recommendations made in this study, the collateral impact of this communicable disease may be partially alleviated. However, if the COVID-19 outbreak continues, medical services will be further negatively affected. Under these circumstances, healthcare systems should adopt other policies and rationally allocate medical resources in order to effectively maintain medical services.

## Figures and Tables

**Table 1:** Clinical variables among 145 adult patients with acute appendicitis during the COVID-19 outbreak.

	Non-Complicated Appendicitis (*N* = 102)	Complicated Appendicitis (*N* = 43)	*p* Value
Age, year, median (IQR)	55.5 (36.8, 65.1)	53.3 (36.4, 65.5)	0.710
Gender			0.360
Female	46 (45.1%)	23 (53.5%)	
Male	56 (54.9%)	20 (46.5%)	
Body mass index, median (IQR)	23.0 (21.3, 25.2)	23.5 (21.0, 25.1)	0.970
Charlson comorbidity index score			0.140
≤2	97 (95.1%)	38 (88.4%)	
>2	5 (4.9%)	5 (11.6%)	
Residence in Taipei/New Taipei City	93 (91.2%)	38 (88.4%)	0.690
Duration of symptoms until presentation to ER, *n* (%)			0.001
≤48 h	93 (91.2%)	33 (79.1%)	
>48 h	9 (8.8%)	9 (20.9%)	
Time from ER visit to surgery (hours), median (IQR)	11.0 (10.0, 14.0)	13.0 (11.0, 15.0)	0.190
Body temperature > 38 degrees Celsius	27 (26.5%)	20 (46.5%)	0.019
White blood count, 10^9^/L, median (IQR)	11.0 (9.0, 14.8)	10.2 (7.9, 11.7)	0.170
Appendectomy performed	100 (98.0%)	36 (83.7%)	0.001
Length of hospital stays (day), median (IQR)	4.0 (3.0, 7.0)	5.0 (3.0, 11.0)	0.080

ER: emergency room.

**Table 2:** Comparison of clinical characteristics between adult patients with acute appendicitis during the COVID-19 epidemic period and the preceding 3-year control period.

	Control Period (*N* = 320)	COVID-19 Period (*N* = 145)	*p* Value
Age, year, median (IQR)	47.9 (33.4, 63.4)	55.2 (36.8, 65.4)	0.088
Gender			0.220
Female	172 (53.8%)	69 (47.6%)	
Male	148 (46.3%)	76 (52.4%)	
Body mass index, median (IQR)	23.0 (21.0, 24.7)	23.1 (21.3, 25.1)	0.210
Charlson comorbidity index score			0.690
≤2	301 (94.1%)	135 (93.1%)	
>2	19 (5.9%)	10 (6.9%)	
Residence in Taipei/New Taipei City	290 (90.6%)	131 (90.3%)	0.920
Duration of symptoms until presentation to ER, n (%)			0.011
≤48 h	291 (90.9%)	120 (82.8%)	
>48 h	29 (9.1%)	25 (17.2%)	
Time from ER visit to surgery (hours), median (IQR)	10.0 (9.0, 13.0)	11.0 (10.0, 14.0)	0.340
Body temperature > 38 degrees Celsius	129 (40.3%)	47 (32.4%)	0.100
White blood count, 10^9^/L, median (IQR)	9.4 (7.6, 11.6)	10.8 (8.9, 13.2)	0.010
Complicated appendicitis	62 (19.4%)	43 (29.7%)	0.014
Length of hospital stays (day), median (IQR)	4.0 (3.0, 6.0)	5.0 (3.0, 8.0)	0.043

ER: emergency room.

**Table 3:** Adjusted multivariate analysis used to predict complicated appendicitis.

Variables	Odds Ratio	95% CI	*p* Value
Age (every one-year increment)	1.01	0.99–1.02	0.245
Male gender (ref: female)	0.93	0.58–1.50	0.793
Body mass index	0.93	0.86–1.01	0.132
Charlson comorbidity index score > 2 (ref: ≤2)	1.38	0.58–3.28	0.459
Residence in Taipei/New Taipei City	0.76	0.40–1.42	0.395
Duration of symptoms until presentation >48 h (ref: ≤48 h)	1.70	1.06–2.36	0.044
Body temperature > 38 degrees Celsius	0.74	0.35–1.57	0.446
White blood counts	1.01	0.76–2.97	0.985
COVID-19 period (ref: control period: 2017–2019)	1.87	1.23–2.52	0.008

**Table 4:** Adjusted multivariate analysis to predict inpatient length of stay.

Variables	Coefficients	95% Confident Interval		*p* Value
		Lower Limit	Upper Limit	
Age (every one-year increment)	0.16	−0.02	0.13	0.524
Male gender (ref: female)	0.64	−1.93	3.21	0.627
Body mass index	−0.15	−0.60	0.31	0.530
Charlson comorbidity index score>2 (ref: ≤2)	2.51	1.25	3.16	<0.001
Residence in Taipei/New Taipei City	−3.05	−7.4	1.29	0.168
Complicated appendicitis	2.10	0.92	3.31	<0.001

## Data Availability

The data are available upon requested from the authors.
